# Haem Oxygenase-1, Ferroptosis and Disorders—A Narrative Review

**DOI:** 10.3390/nu17243921

**Published:** 2025-12-15

**Authors:** James Pham, Michel Refesse, Ashwa Saeed, Gladys O. Latunde-Dada

**Affiliations:** 1 Department of Nutritional Sciences, School of Life Course and Population Sciences, King’s College London, Franklin-Wilkins-Building, 150 Stamford Street, London SE1 9NH, UK; james.pham@kcl.ac.uk (J.P.); michel.refesse@kcl.ac.uk (M.R.); ashwa.saeed@kcl.ac.uk (A.S.); 2 Faculty of Sciences and Technology, Engineering School (ESIROI), University of Reunion Island’s, Agri-Food, 97410 Saint-Pierre, Reunion Island, France

**Keywords:** haem oxygenase, ferroptosis, iron, cancer, degenerative disease

## Abstract

Ferroptosis is a non-apoptotic form of cell death that is driven by iron and reactive oxygen species (ROS). This process is characterised by lipid peroxidation, which damages cell membranes and distinguishes it from other types of cell death. Excess iron promotes ferroptosis through Fenton chemistry, leading to increased ROS production. While glutathione peroxidase 4 has been identified as a key regulator of this process, other factors, such as the ferroptosis suppressor protein 1 (FSP1), suggest that regulation is more complex. Ferroptosis has been associated with several degenerative diseases, including Alzheimer’s disease, Parkinson’s disease, acute kidney injury, liver disorders, and cancer. The enzyme haemoxygenase-1 (HO-1) plays dual roles: it can promote ferroptosis by releasing iron or provide protection through its antioxidant effects in various organs and tissues. HO-1 increases iron levels through the catabolism of haem which can heighten sensitivity to ferroptosis by influencing iron trafficking and ferritin expression. Conversely, HO-1 has demonstrated nephroprotective effects in cases of renal injury and other disorders. HO-1′s involvement in regulating iron metabolism and its antioxidant capabilities can lead to differing outcomes, highlighting key players in the ferroptosis process. The Nrf2/HO-1 axis is crucial for its antioxidant properties in various disorders. Moreover, dietary sources can enhance HO-1 induction through Nrf2 regulation. Hence, HO-1 acts as both a modulator and a mediator, presenting new therapeutic targets for cancer, neurodegeneration, and kidney and liver diseases.

## 1. Ferroptosis

Cell death is an important mechanism essential for the proper development and homeostasis of tissues in multicellular organisms. While there are instances where cells are unable to control this process (e.g., necrotic cell death), nature has evolved several regulated forms of cell death, including ferroptosis [1]. In the 1950s and 1960s, Harry Eagle discovered that mammalian cells in culture would die if they did not obtain enough cysteine, while cells able to make their own cysteine survived [2]. These observations hinted at ferroptosis, showing that having enough cysteine and glutathione is important for controlling a regulated type of oxidative cell death [2]. Ferroptosis was first described by [3] Dixon et al. as non-apoptotic oxidative cell death dependent on iron and reactive oxygen species (ROS) [4]. This process, characterised by lipid peroxidation-mediated membrane damage [5], differs from other forms of cell death, such as apoptosis, autophagy, and necroptosis, in terms of biochemistry, morphology, and genetics [3,6]. Morphologically, ferroptotic cells exhibit reduced mitochondrial size, increased membrane density, and a lack of chromatin condensation, distinguishing ferroptosis from other forms of cell death like apoptosis and necroptosis [7].

The overabundance of iron facilitates ferroptosis through Fenton chemistry, contributing to ROS production [8]. Redox-active iron from the cytosolic labile iron pool (LIP) reacts with hydrogen peroxide (H_2_O_2_), creating hydroxyl free radicals [9]. Superoxides then reduce ferric iron (Fe(III)) back to ferrous iron (Fe(II))and oxygen in Haber–Weiss reactions [4]. Normally, excess Fe is sequestered into ferritin, an iron storage protein composed of ferritin light chain (FTL) and ferritin heavy chain (FTH1) [7], which can safely hold from a few hundred to 4500–5000 iron atoms within its core [10]. The heavy chain (FTH1) has ferroxidase activity, converting Fe^2+^ into Fe^3+^ for safe storage, while the light chain (FTL) mainly helps mineralise and organise iron inside the ferritin core [11]. Conversely, under oxidative stress, excess superoxides liberate Fe(II) from iron-containing compounds, such as ferritin and haem accumulating ROS involved in membrane lipid peroxidation and ferroptotic cell death [4].

### Induction of Ferroptosis Occurs via Two Main Mechanisms

(i) Inhibition of a specific light-chain subunit of system Xc- (cystine–glutamate antiporter), named solute carrier family 7 member 11 (SLC7A11):
  – Inhibitors, such as erastin and sorafenib, or high extracellular glutamate concentrations inhibit cystine uptake via SLC7A11; therefore, less cysteine combines with methionine, resulting in decreased glutathione (GSH) synthesis, where GSH is a cofactor of GPX4 [1]. These compounds are part of the class I ferroptosis inducers, which act by depleting intracellular cysteine and impairing GPX4 function [12]. Reduced GPX4 activity results in the accumulation of lipid hydroperoxides, characteristic of ferroptotic cell death [13,14].
(ii) Inactivation of glutathione peroxidase 4 (GPX4) directly via GPX4 inhibitors (e.g., RAS-selective lethal small molecule 3; RSL3):
  – Under normal circumstances, GPX4 oxidises GSH to GSSG and reduces toxic lipid peroxides (L-OOH) to lipid alcohols (L-OH) [7]. However, following the inhibition of GPX4 activity, the accumulation of ROS from lipid peroxidation consequently leads to ferroptosis, irrespective of GSH depletion [15]. While GPX4 has long been considered the central regulator of ferroptosis, recent studies have identified alternative pathways that bypass GPX4. For instance, FSP1 (ferroptosis suppressor protein 1) acts independently by regenerating CoQ10, a lipid-soluble antioxidant, thereby preventing lipid peroxidation. This discovery expands the therapeutic landscape beyond GPX4-centric strategies [16].
  – PUFAs (e.g., arachidonic acid) or phospholipids with polyunsaturated acyl tails (PL-PUFAs) are the main substrates of ferroptotic lipid peroxidation, generated by Acyl-CoA Synthetase Long-Chain Family Member 4 (ACSL4) and Lysophosphatidylcholine Acyltransferase (LPCAT) enzymes that incorporate free PUFAs into lipid membranes [17]. Recent studies have revealed that the specific composition of membrane phospholipids, particularly the abundance of polyunsaturated fatty acids (PUFAs), plays a decisive role in ferroptosis sensitivity. Cells enriched in PUFA-containing phospholipids are more prone to lipid peroxidation, especially when antioxidant defences such as GPX4 or FSP1 are compromised. This lipidomic signature is now considered a predictive marker for ferroptosis vulnerability across various cell types [18]. Hence, unlike apoptosis or other non-ferroptotic oxidative injuries, ferroptosis exhibits a unique biochemical signature defined by the accumulation of redox-active iron, enhanced Fenton chemistry, and excessive lipid hydroperoxide formation. This process is accompanied by a failure of key antioxidant defence systems, most notably the glutathione/GPX4 axis, as well as the CoQ10 pathway. Moreover dietary constituents, including naturally occurring antioxidants and molecules that modulate iron handling, can influence oxidative pressure and iron availability within tissues. By affecting these two central drivers of ferroptotic cell death, dietary patterns and specific bioactive compounds may indirectly contribute to the regulation of ferroptosis in physiological and pathological contexts.

## 2. Haem Oxygenase-1 (HO-1)

Enzymatic degradation of haem is a potential source of iron on which ferroptosis is dependent. The enzyme involved in this reaction is HO-1, which is a highly inducible enzyme [19]. Degradation of haem by HO-1 leads to the formation of biliverdin, carbon monoxide (CO) and Fe(II) [20]. Recent findings have expanded the role of HO-1 beyond haem degradation, highlighting its influence on intracellular iron trafficking and ferroptosis sensitivity. In particular, HO-1 has been shown to regulate the expression of iron transporters such as divalent metal transporter (DMT1) and ferroportin, which control iron influx and efflux across cellular membranes. This modulation affects the size and reactivity of the labile iron pool (LIP), a key determinant of ferroptosis susceptibility [21].

### HO-1: A Double-Edged Sword in Ferroptosis

A large body of evidence suggests that HO-1 induction plays beneficial roles in several pathological conditions. Despite strong evidence from these studies demonstrating the cytoprotective effects of HO-1 through haem degradation, the mechanisms underlying the beneficial actions of HO-1 are not yet clearly defined [22]. The cytoprotection of HO-1 is possibly exerted through haem catabolism end-products [23]. For instance, biliverdin that is produced is converted into bilirubin by the enzyme biliverdin reductase, both of which are involved in scavenging ROS [24]. CO, on the other hand, increases cyclic guanosine monophosphate (cGMP) generation via soluble guanylate cyclase (sGC) and also exerts its effects on other signalling pathways such as mitogen-activated protein kinase (MAPK) pathways to elicit tissue protection, through anti-apoptotic, anti-inflammatory, anti-proliferative, and pro-angiogenic effects [24]. In addition, the release of free iron during haem catabolism induces ferritin expression, which further contributes to antioxidant defence and cytoprotection [25].

However, the fate of iron that is released through the process of haem degradation has been thought to be controversial because while iron is essential to the functioning of important proteins, it has been proven to be toxic in occasions where it participates in uncontrolled redox reactions [26]. Multiple studies attribute the cytoprotective effects of HO-1 to the increased expression of ferritin and its iron-sequestering effects, which therefore decreases the intracellular pool of iron. Moreover, ferroxidase activity of heavy-chain ferritin oxidises Fe (II) to Fe (III), which is catalytically inactive, thus acting as a mechanism to resist redox reactions that contribute to oxidative damage [27]. Experimental evidence supportive of this hypothesis has revealed that cells overexpressing HO-1, resistant to H_2_O_2_-induced cell death, contain lower freely unbound iron levels despite higher total intracellular iron levels compared to wild-type cells [28]. This discrepancy is likely explained by compensatory increases in ferritin (heavy chain) synthesis, transferrin receptor expression and haem synthesis also observed in HO-1-overexpressing cells [28].

On the other hand, HO-1 activity in some instances has been shown to increase intracellular iron levels despite the increase in ferritin [29] (yet recent studies indicate that this rise in iron is often counterbalanced by a concomitant induction of ferritin and ferroportin, which sequester or export excess iron and thereby preserve the cytoprotective effects of HO -1) [30]. Furthermore, ref. [19] Otterbein et al. demonstrated through in vivo investigation that overexpression of HO-1 through intratracheal administration of recombinant HO-1 did not increase ferritin light chain and ferritin heavy chain expression. Other studies have also reported that HO-1 overexpression, unless from exogenous hemin, did not further increase baseline ferritin synthesis, indicating that haem levels regulate haem catabolism and that the cytoprotection offered by HO-1 may not be due to significant levels of haem degradation [31]. The controversy surrounding the effects of free iron released as a result of HO-1 overexpression has been resolved by [29] Sutter and Dennery, who found that it was beyond a certain HO-1 overexpression threshold (15-fold HO-1 activity) that the iron released via haem degradation augmented oxidative damage and that cytoprotective effects were observed at low to moderate levels of HO-1 expression.

**Figure 1:**
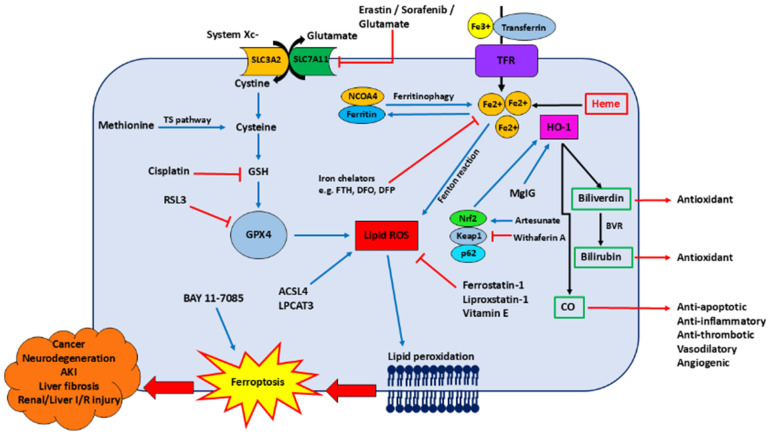
The mechanisms of ferroptosis involving haem oxygenase-1 (HO-1). Inhibition of solute carrier 7 family member 11 (SLC7A11), part of system Xc-, reduces intracellular cysteine levels, leading to glutathione (GSH) depletion and inactivation of glutathione peroxidase (GPX4). Methionine can also be converted to cysteine via the transsulfuration (TS) pathway. Ferroptosis inhibitors, such as cisplatin and ras-selective lethal small molecule 3 (RSL3), can inhibit GSH levels and GPX4, respectively. GPX4 inactivation results in accumulating ROS levels, lipid membrane peroxidation, and ferroptosis. Other contributors of ROS accumulation include the enzymes acyl-CoA synthetase long-chain family member 4 (ACSL4) and lysophosphatidylcholine acyltransferase (LPCAT), providing polyunsaturated fatty acids as the main substrates for lipid peroxidation, and redox-active iron (Fe (II)) catalysing the Fenton reaction. The pool (LIP) of Fe(II) is derived from 3 main sources: (1) extracellular Fe(III)–transferrin complex uptake into the cell via the transferrin receptor (TFR); (2) haemcatabolism catalysed by HO-1; (3) nuclear receptor coactivator 4 (NCOA4)-mediated ferritinophagy. Iron chelators, such as ferritin heavy chain (FTH), deferoxamine (DFO), and deferiprone (DFP), can minimise cellular Fe(II) levels. HO-1 expression is mediated by the p62-Kelch-like ECH-associated protein 1 (KEAP1)–Nuclear factor erythroid 2-related factor 2 (Nrf2) pathway, and by molecules such as magnesium isoglycyrrhizinate (MgIG). The cytoprotection of HO-1 is likely offered by its by-products; (1) bilirubin is converted into biliverdin by biliverdin reductase, in which both bile pigments show antioxidant activity; (2) carbon monoxide (CO) helps with the promotion of anti-apoptotic molecules, generation of anti-inflammatory cytokines, prevention of thrombosis, blood vessel vasodilation, and angiogenesis; (3) Fe(II)-stimulated ferritin synthesis sequesters iron and reduces the associated pro-oxidant activity. Overall, HO-1-mediated ferroptosis is implicated in numerous diseases, including cancer, neurodegenerative diseases, acute kidney injury (AKI), liver fibrosis, and renal/hepatic ischemia/reperfusion (I/R) injury.

The growing body of evidence highlights the importance of HO-1 in ferroptosis given the role of HO-1 in providing iron on which ferroptosis is dependent [32,33,34,35,36]. More importantly, HO-1 and ferroptosis have been implicated in a multitude of pathologies, including neurodegeneration, cancer, acute kidney injury and liver damage [13,32,37,38,39]. Therefore, this review aims to explore the role of HO-1 and ferroptosis in such diseases. Figure 1 illustrates the key molecular pathways regulating ferroptosis, highlighting the central roles of iron metabolism, lipid peroxidation, and antioxidant defence mechanisms.

## 3. Cancer

### 3.1. Ferroptosis and Cancer

Cancer is characterised by abnormal cell growth which necessitates cell death to overcome the hyperproliferative nature of the disease [40]. Moreover, cancer cells contain high levels of iron as a result of disrupted iron homeostasis to facilitate the provision of ribonucleotide reductase, an iron-dependent enzyme essential for the growth of cancer cells [41]. While excessive iron has been associated with oxidative damage, cancer cells resist this damage due to the high levels of antioxidants such as glutathione [42]. Ferroptosis is therefore relevant as a therapeutic in cancer and has been identified as a novel target in cancer treatment. This is especially important given that the detrimental genetic alterations associated with cancer have been shown to suppress alternative forms of cell death pathways such as apoptosis [13]. Ferroptosis inducers have therefore been extensively researched. One such study was conducted by [3] Dixon et al., who found that treating fibrosarcoma cells with erastin, a ferroptosis inducer, led to a time-dependent increase in ROS which eventually resulted in cell death and cancer suppression. Furthermore, when cells were treated with deferoxamine, which is an iron chelator, the build-up of ROS and consequent cell death were suppressed. Further research has also found that erastin had similar effects of inducing ferroptosis in human cancer cell lines other than fibrosarcoma cells (43B, BJeHLT, BJeLR and Calu-1) [43]. More recently, Wei et al. (2023) [44] demonstrated in cervical cancer cells that erastin activates the Nrf2/HO-1 pathway, and that silencing HO-1 restores ferroptotic sensitivity, while Li et al. (2022) confirmed a similar protective role of Nrf2/HO-1 in ovarian cancer [44,45]. This however is not the case in all cancer cell lines, and recent studies have demonstrated that the sensitivity of non-small-cell lung cancer (NSCLC) cell lines to erastin can be increased by combining erastin with acetaminophen, a drug that when used together with erastin has previously been shown to elicit cell death in a similar way to erastin-induced ferroptosis [46].

### 3.2. Modulation of HO-1 in Ferroptosis and Cancer

HO-1 expression is increased in numerous cancer cell lines [47]. It has been postulated that HO-1 and corresponding metabolites, hemin and CO-releasing molecules (CORMs), play a crucial role within ferroptosis in cancer cells [32]. Exposing HT-1080 fibrosarcoma cells to erastin and HO-1 by-products increased HO-1 expression, lipid peroxidation and rate of ferroptotic cell death, yet interestingly, such outcomes were not associated with biliverdin and bilirubin despite the cytoprotective mechanisms of these bile pigments aforementioned [24,32]. BAY 11-7085, which inhibits NF-KB phosphorylation, induces ferroptotic cancer cell death through HO-1 mediation, promoting labile Fe(II) accumulation and perturbations in redox balance [33]. Recent studies confirm this mechanism, showing that BAY triggers ferroptosis via the Nrf2–SLC7A11–HO-1 axis, where HO-1 upregulation drives iron release and lipid peroxidation [33]. This provides further evidence to demonstrate the significance of HO-1 as a modulator of ferroptosis.

### 3.3. HO-1-Mediated Ferroptosis Induced by Small Molecules and Natural Compounds

Interestingly, it was also observed that inhibition of SLC7A11 amplified BAY-induced ferroptosis, exacerbating cell death [33]. Similarly, another ferroptosis inducer, Withaferin A (WA), significantly upregulated HO-1 activity, leading to elevated free intracellular Fe(II) and subsequent ferroptosis in resistant neuroblastoma cell lines [34]. Impressively, ex vivo administration of WA showed significant tumour regression and no relapse post-termination of WA treatment [34]. Furthermore, WA-encapsulated nanoparticles were then investigated in vivo and suppressed tumour growth was observed without adverse weight loss [34].

Studies that examined artesunate, a typical anti-malarial drug which is also used in anti-cancer treatment for its ability to cause ROS-dependent ferroptotic cell death, showed that treatment with the drug killed head and neck cancer (HNC) cells in a discriminatory fashion without affecting normal cells [35]. The mechanisms by which artesunate enabled this process were attenuating intracellular GSH and augmenting ROS levels necessary for ferroptosis [35]. Although artesunate is capable of inducing ferroptosis, reduced sensitivity to artesunate was found in HNC cells that are resistant to cisplatin (a chemotherapeutic agent) due to Nrf2 activation. Thus, genetic silencing of the Nrf2-HO-1 pathway was found to alleviate the resistance to artesunate-induced ferroptosis in cells with reduced sensitivity to artesunate [35]. More recently, artesunate has also been shown to induce ferroptosis in hepatocellular carcinoma when delivered via nanoparticles, enhancing ROS accumulation, Fe(II)release and lipid peroxidation, thereby improving therapeutic efficacy in vivo [48].

### 3.4. Anti-Ferroptotic Actions of HO-1 in Cancer

HO-1 also elicits anti-ferroptotic effects in hepatocellular carcinoma (HCC) cells since Nrf2 activation inhibits ferroptosis via p62-mediated Keap1 degradation, upregulating the actions of Nrf2 on its target genes, HO-1 and FTH1 [36]. In vitro and in vivo investigations demonstrate that inhibiting Nrf2 increases susceptibility of HCC cells to ferroptosis; therefore, both findings illuminate the p62-Keap1-Nrf2 pathway as a therapeutic target in ferroptosis [36]. Similar anti-ferroptotic effects are found in A459 lung adenocarcinoma cells with elevated HO-1 expression, leading to increased ferritin synthesis for iron binding and reduction in the associated ROS activity [28]. More recent research using bioinformatics analysis and microarray data of differentially expressed genes (DEGs), identified transcription factor AP-2 alpha (TFAP2A) as a mediator of ferroptosis via Nrf2 pathways in gallbladder carcinoma [49]. Ferroptosis is an ongoing area of research where emerging evidence is identifying novel targets of ferroptosis as potential candidates for cancer therapy. Given the multimodal approach to cancer treatment, such research helps develop our understanding of how ferroptosis can be modulated to suit specific cell lines for effective treatment strategies.

## 4. Neurodegenerative Diseases

### HO-1 and Alzheimer’s Disease and Parkinson’s Disease

Alzheimer’s disease (AD) and Parkinson’s disease (PD) are characterised by classical features of ferroptosis, including oxidative stress and dysregulated iron metabolism. In AD, iron is mainly found in astrocytes and neurons surrounding amyloid plaques, whereas in PD, it is localised in glial cells and neurons. Cerebellar granule neurons from transgenic mice overexpressing Hmox1 protect against H_2_O_2_- and glutamate-mediated neuronal cell death and decreased ROS generation [50]. Neuroprotective effects observed in HO-1 overexpression are similarly found in selective astrocytes, which are necessary for the maintenance of the blood–brain barrier, in mouse models of intracerebral haemorrhage (ICH) [51]. Such benefits include reduced mortality, blood–brain barrier disruption, and neuronal loss in astrocytes post-ICH [51]. More recently, HO-1 has been implicated in ferroptosis-related mechanisms in neurodegeneration. Studies on vascular dementia highlighted that HO-1 regulates iron metabolism and ferroptosis, protecting neurons by mitigating oxidative stress, reducing inflammation, and enhancing mitochondrial function [21]. Modulating HO-1) aims to reduce oxidative stress and damage caused by amyloids in AD and to prevent ferroptosis and the degeneration of dopaminergic neurons in PD. Therefore, using HO-1 inhibitors or iron chelators in AD may help alleviate oxidative damage and amyloid toxicity. In PD, targeting HO-1 with ferroptosis inhibitors (such as ferrostatin-1 and liproxstatin-1), iron chelators, antioxidants, and Nrf2 inducers (like sulforaphane and curcumin) may protect dopaminergic neurons. These need to be based on context-specific short-term induction of HO-1 for antioxidant benefits versus long-term inhibition to prevent iron overload.

## 5. Kidney Disease

### 5.1. Ferroptosis and Acute Kidney Injury

Acute kidney injury (AKI), once known as acute renal failure (ARF), is a disease condition that occurs as a result of various conditions such as ischemia, obstructions in the urinary tract, nephrotoxic drugs, etc., and its pathogenic mechanisms have been attributed to vasoconstriction, oxidative stress and cell death pathways such as apoptosis and ferroptosis [52]. Research has found ferroptosis to be of significance in the death of parenchymal cells of the renal tubules, a key pathological cause of acute kidney injury [53]. Thus, targeting ferroptosis as a novel form of treatment in acute kidney injury becomes particularly relevant, especially due to treatment options being limited. Recent reviews emphasise that renal tubular cells are among the most ferroptosis-vulnerable tissues, making ferroptosis inhibition a promising therapeutic [54].

### 5.2. Emerging Molecular Targets Regulating Ferroptosis in AKI

An alternative ferroptosis therapeutic target in AKI involves Pannexin 1 (Panx1), an ATP-releasing channel protein involved in apoptosis [55]. Importantly, deletion of Panx1 improved renal injury in ischaemia/reperfusion, (I/R)-treated mice through decreased ferroptosis-related lipid peroxidation and iron accumulation [55]. Similar results were found in vitro along with a decreased expression of ferroptosis-related proteins, NCOA4 and FTH1 [55]. These results were concomitant with upregulated HO-1 expression in Panx1-/- mice, overall suggesting that the protective benefits of Panx1 deletion are mediated by the MAPK/ERK pathway to decrease nuclear receptor coactivator 4 (NCOA4)-mediated ferritinophagy and increase HO-1 expression [55].

However, ferroptosis is not the only regulated cell death process occurring in AKI as synergistic crosstalk between ferroptosis and necroptosis is present in murine I/R injury [56]. This is supported by the fact that combination therapy can be more effective in treating acute renal damage [53,57]. Ferroptosis has also been implicated in rhabdomyolysis-induced kidney injury, another type of AKI, characterised by myoglobin cylinder formation [58]. While it was previously thought that the accumulation of myoglobin and its subsequent deposition in the kidney are the main mechanisms behind kidney damage, recent research indicated a role of Fe (II) released as a product of myoglobin metabolism and its ability to drive lipid peroxidation as a crucial mechanism of rhabdomyolysis-induced kidney injury [59]. Furthermore, when deferoxamine, an iron chelator, was injected into rats with induced renal failure, a significant alleviation in rhabdomyolysis-induced kidney injury was observed [60]. Moreover, ref. [61] Guerrero-Hue et al. found that treatment with curcumin, an antioxidant molecule, protected against renal dysfunction associated with rhabdomyolysis.

Collectively, the existing literature justifies the interaction between ferroptosis and AKI and suggests that the inhibition of ferroptosis may provide cytoprotection in particular cell lines. Many recent studies highlight the mechanisms of ferroptosis and provide prospective candidates for novel therapies in acute renal failure.

### 5.3. HO-1 and AKI

Initial evidence revealing that renoprotection is mediated by HO-1 was first discovered by [62] Nath and colleagues in rhabdomyolysis-induced AKI and then in cisplatin nephrotoxicity [63]. Subsequently, the nephroprotective effects of HO-1 were evidenced in renal I/R injury [64,65]. Bolisetty, Zarjour & Agarwal [63,66,67] reviewed the plethora of evidence involving global or kidney-specific HO-1 deficiency or overexpression, confirming the cytoprotection conferred by HO-1 during AKI through mechanisms such as oxidative stress, apoptosis and inflammation. HO-1 also mediates protection co-dependently with H-ferritin (FtH) expression, as ablation of FtH in the proximal renal tubule intensifies structural and functional damage in AKI, irrespective of significantly increased HO-1 expression [67]. It was also found that curcumin exerted its protective effects through the activation of HO-1 which reduced myoglobin-mediated oxidative stress and ferroptosis associated with rhabdomyolysis-induced AKI [61].

Further research delving into the role of HO-1 in the ferroptotic death of renal proximal tubules has found that whilst HO-1 is upregulated in the renal proximal tubules following AKI, ferroptosis was augmented in HO-1-deficient mice [68]. This suggests that iron released as a result of HO-1-catalysed reactions does not directly mediate ferroptosis and instead has anti-ferroptotic effects in the case of renal epithelial cells [68]. Consistently, it was shown that modulation of the Keap1/Nrf2/HO-1 axis protects against endotoxin-associated AKI by alleviating ferroptosis and lipid peroxidation, highlighting HO-1 as a key checkpoint in ferroptotic regulation [69].

HO-1 has been additionally demonstrated to mitigate AKI in hemin-treated mice, as attested by attenuated inflammatory and oxidative stress biomarkers by hemin-induced HO-1 following renal I/R injury [70]. Nevertheless, the scarcity of research studying the regulation of HO-1 explicitly in ferroptotic cell death in acute renal failure and I/R injury warrants further investigation.

In summary, the extensive evidence strongly supports the protective properties of pharmacological induction of HO-1 in animal and cell models of acute renal failure [63,66,67,71]. Moving forward, it is essential to determine the safety and effectiveness of HO-1 induction, particularly regarding treatment duration and optimal levels of upregulation. This needs to be coupled with the implementation of translational research focused on applying HO-1 targets in human AKI therapy. Moreover, it is important to balance HO-1′s cytoprotective antioxidant role with the potential risk of increasing labile iron levels if induction is excessive [69].

## 6. Liver Disease

The most significant component in the pathogenesis of liver disease involves cell death pathways [72]. The liver is prone to iron toxicity given its role in storing excess iron from the bloodstream in hepatic parenchymal cells [73]. Consequently, ferroptosis, being an iron-dependent form of cell death, has been implicated in the pathogenesis of various liver diseases [72]. Recent reviews emphasise that ferroptosis contributes not only to hepatocyte injury but also to hepatic stellate cell activation, thereby linking it to both acute and chronic liver injury, fibrosis, and hepatocarcinogenesis [74].

### 6.1. HO-1 and Liver Damage

Despite the lack of evidence for the role of HO-1 in ferroptosis during acute liver damage, HO-1 has been shown to exert its protection through other modalities of cell death, including apoptosis. Some evidence suggests that HO-1 mediates its pro-apoptotic effects through indirect pathways rather than through its metabolites. For example, HO-1 can inhibit the proliferation of hepatic stellate cells (HSCs), modulate the expression of apoptosis-related proteins, in particular caspase-3 and Bcl-2, and downregulate the NF-KB pathway and downstream inflammatory factors to augment apoptotic HSC death [75,76]. Other studies show that HO-1 prevents hepatic failure induced by Propionibacterium acnes and lipopolysaccharide (LPS) by modulating adaptive immune responses through suppressing the maturation of dendritic cells, hindering the activation, proliferation, and Th1 polarisation of CD4+ T cells necessary to infiltrate the liver and contribute to liver injury, and attenuating pro-inflammatory cytokine production [77].

Nevertheless, substantial evidence exists for HO-1-induced hepatic protection via haem degradation by-products. The mechanism by which CO confers protection against liver I/R injury in an ex vivo liver perfusion model was through the p38 MAPK signalling pathway, independent of cGMP, and interestingly, endogenous CO can be substituted with exogenous CO to mitigate I/R insult [78]. Haem-derived iron also shows protection via stimulating H-ferritin expression which is associated with the protection of rat livers from I/R injury [79]. However, the mechanisms underpinning the cytoprotective effects of haem iron-induced H-ferritin remain unknown.

On the contrary, inhibiting HO-1 expression by the Nrf2/Keap pathway could lower hepatic iron accumulation and CO levels and ameliorate liver fibrosis [80]. This is conflicted by the evidence, including the previous studies mentioned, whereby the induction of HO-1 is beneficial in suppressing liver fibrosis in terms of inflammation [81]. Thus, future research to clarify the independence of the many putative actions of HO-1 in liver fibrosis and I/R injury concerning the direct involvement in ferroptosis is warranted.

### 6.2. Ferroptosis in Acute Liver Failure (ALF)

Ferroptosis also has deleterious effects on acute liver failure (ALF). Treatment of glycyrrhizin, an inhibitor of the high-mobility group protein B1 (HMGB1) which is an inflammatory factor associated with the pathology of ALF, mitigated acute liver damage and inhibited ferroptosis by stimulating the Nrf2/HO-1/HMGB1 pathway and increasing GPX4 levels [82]. The ferroptosis regulator GPX4 is essential for hepatocyte survival and optimal hepatic function, and with a vitamin E-supplemented diet (ferroptosis inhibitor), it can abrogate hepatocyte degeneration from lipid peroxidation [83]. Conversely, in mice, GPX4 knockout resulted in significant lipid peroxidation and death, which was exacerbated by vitamin E deficiency [83]. This is supported by comparable results found in endothelial cell depletion of GPX4 and vitamin E [84]. These findings are consistent with new data showing that ferroptosis-linked GPX4 deficiency sensitises hepatocytes to oxidative stress and accelerates ALF progression [85].

Overdosing on acetaminophen (APAP, commonly known as paracetamol) is one of the leading causes of ALF [8]. Ferroptosis induction has been evidenced in APAP-treated primary mouse hepatocytes and the inhibition of ferroptosis by ferrostatin-1 consequently inhibited APAP-induced cell death [86]. However, these inhibitory effects occurred independently of decreased acetaminophen metabolism or GSH depletion [86]. Recent findings confirm the role of ferroptosis in driving APAP-induced liver cell death. They highlight the mechanisms of lipid peroxidation, primarily involving the n-6 polyunsaturated fatty acid arachidonic acid, which undergoes auto-oxidation during ferroptosis, ultimately leading to APAP-induced hepatotoxicity [87]. Overall, these studies corroborate the favourable effects of ferroptosis induction in reducing HSC activation to attenuate liver fibrosis and the role of ferroptosis in the progression of other liver diseases such as non-alcoholic fatty liver disease (NASH) hepatic I/R injury and acute liver failure. Manipulation of HO-1, and ferroptosis, therefore, has promising potential in the treatment of liver and other diseases (Table 1).

## 7. Natural Food Inducers of HO-1 and Antioxidant Function

Capsaicin; resveratrol from grapes, cocoa, peanuts, berries, and wine; isothiocyanates from broccoli; organosulfur compounds in garlic; quercetin; and curcumin are examples of natural dietary sources that have been shown to enhance HO-1 induction mostly via Nrf2 transcriptional regulation in cells. For example, ferulic acid is a bioactive polyphenol found in various foods, including cereals such as wheat [88]. It has been shown to exhibit antioxidant properties by activating the Nrf2 signalling pathway [89]. Moreover, studies in MIN6 cells have demonstrated that ferulic acid increases the expression of HO-1, possibly through Nrf2 activation, which helps suppress erastin-induced ferroptosis [90]. Similarly, epigallocatechin gallate (EGCG) from green tea is also recognised for its antioxidant properties [91], which are conferred by HO-1 through the Nrf2 signalling pathway. The signal pathways that activate Nrf2 upregulate HO-1, which can suppress NF-κB signalling by downregulating IKK/NF-κB. This process has both antioxidant and anti-inflammatory effects. Moreover, Bach1 directly binds to the Maf recognition elements (MAREs) in the HO-1 gene promoter, thereby inhibiting its transcription. Other natural inducers of HO-1 include haem and analogs, polyphenols such as curcumin (from Curcuma longa), quercetin (a flavonoid found mainly in fruits and vegetables, such as red onions and tea leaves), gallic acid from Allium sativum (garlic), and anthocyanins from fruits like blackberries, raspberries, and strawberries [92]. The effectiveness of these plant-based HO-1 inducers in target cells, organs, and tissues depends on their absorption, which can vary significantly in the gastrointestinal tract. Other important factors include the concentrations of these compounds, their stability, and the metabolites produced during systemic metabolism.

## 8. Conclusions

Ferroptosis is non-apoptotic oxidative cell death dependent on iron and reactive oxygen species (ROS), distinct from apoptosis. It is involved in several diseases, where it presents a dual therapeutic role. In cancer, its induction targets apoptosis-resistant cells, whereas in neurodegenerative, renal, and hepatic disorders, its inhibition offers a protective strategy. HO-1 plays a complex role, modulating ferroptosis sensitivity through iron metabolism. Understanding these mechanisms thus opens innovative therapeutic perspectives for numerous diseases, making the ferroptotic pathway a key target for future biomedical research. Consequently, pharmacological inducers and inhibitors of HO-1 such as hemin and ZnPP modulators, as well as ferroptosis inhibitors such as ferrostatin-1, are being studied for clinical applications. The dual role of HO-1 complicates clinical trials; therefore, indirect approaches involving Nrf2 activators or natural compounds are emerging. Dietary food types also promote the induction of HO-1 primarily through Nrf2 transcriptional regulation in cells and tissues.

## Figures and Tables

**Table 1:** Haem oxygenase, oxidative stress and ferroptosis.

Disorders	Effects	References
Cancer	Erastin induction activates the Nrf2/HO-1 pathway in cervical and ovarian cancer cells.	[44,45]
	BAY induces ferroptosis through the Nrf2–SLC7A11–HO-1 axis; HO-1 upregulation promotes iron release and lipid peroxidation.	[33]
	Withaferin A (WA) induces ferroptosis by strongly upregulating HO-1, increasing free intracellular Fe(II), and triggering ferroptotic cell death.	[34]
	Elevated HO-1 expression in lung adenocarcinoma exerts anti-ferroptotic effects by increasing ferritin synthesis for iron sequestration and reducing ROS activity.	[28]
Neurodegenerative diseases	HO-1 regulates iron metabolism and ferroptosis, protecting neurons by mitigating oxidative stress, reducing inflammation, and enhancing mitochondrial function. Neuroprotective effects observed in HO-1 overexpression are similarly found in selective astrocytes.	[21,51]
Intracerebral haemorrhage (ICH)	HO-1 overexpression shows neuroprotective effects in selective astrocytes, which are essential for maintaining the blood–brain barrier in mouse ICH models.	[51]
Acute kidney injury (AKI)	HO-1 demonstrates nephroprotective effects in renal ischemia/reperfusion injury.	[64,65]
	HO-1 mediates protection in coordination with H-ferritin (FtH); FtH ablation in proximal tubules worsens structural and functional kidney damage despite elevated HO-1 expression.	[67]
	Hemin-induced HO-1 mitigates AKI in mice by reducing inflammatory and oxidative stress biomarkers following renal ischemia/reperfusion injury.	[70]
Liver disease	HO-1 inhibits hepatic stellate cell (HSC) proliferation, modulates apoptosis-related proteins (caspase-3, Bcl-2), and downregulates the NF-κB pathway and related inflammatory factors, thereby promoting apoptotic HSC death.	[75,76]
	ALR mitigates acute liver damage and inhibits ferroptosis by activating the Nrf2/HO-1/HMGB1 pathway and increasing GPX4 levels.	[82]

## Data Availability

This is a narrative review article and there is no data to share.

## Abbreviations

ROS, reactive oxygen species; GPX4, glutathione peroxidase 4; FSP1, ferroptosis suppressor protein 1; HO-1, haem oxygenase-1; LIP, labile iron pool; FTL, ferritin light chain; FTH1, ferritin heavy chain; H_2_O_2_, hydrogen peroxide; Fe(II), ferrous iron; Fe(III), ferric iron; SLC7A11, solute carrier family 7 member 11; GSH, glutathione; GSSG, oxidised glutathione; L-OOH, lipid hydroperoxides; L-OH, lipid alcohols; CoQ10, coenzyme Q10; ACSL4, acyl-CoA synthetase long-chain family member 4; LPCAT, lysophosphatidylcholine acyltransferase; PUFAs, polyunsaturated fatty acids; PL-PUFAs, phospholipid-PUFAs; CO, carbon monoxide; DMT1, divalent metal transporter 1; MAPK, mitogen-activated protein kinase; ERK, extracellular signal-regulated kinase; cGMP, cyclic guanosine monophosphate; sGC, soluble guanylate cyclase; TFR, transferrin receptor; NCOA4, nuclear receptor coactivator 4; DFO, deferoxamine; DFP, deferiprone; Nrf2, nuclear factor erythroid-2–related factor 2; KEAP1, Kelch-like ECH-associated protein 1; MgIG, magnesium isoglycyrrhizinate; HMGB1, high-mobility group box-1; AKI, acute kidney injury; I/R, ischemia/reperfusion; Panx1, pannexin-1; HSC, hepatic stellate cell; LPS, lipopolysaccharide; APAP, acetaminophen; ALF, acute liver failure, NASH, non-alcoholic fatty liver disease.
